# Differential influence of arterial blood glucose on cerebral metabolism following severe traumatic brain injury

**DOI:** 10.1186/cc7711

**Published:** 2009-02-06

**Authors:** Monika Holbein, Markus Béchir, Silke Ludwig, Jutta Sommerfeld, Silvia R Cottini, Marius Keel, Reto Stocker, John F Stover

**Affiliations:** 1 Surgical Intensive Care Medicine, University Hospital Zuerich, Raemistrasse 100, Zuerich, 8091, Switzerland; 2 Department of Surgery, Division of Trauma Surgery, University Hospital Zuerich, Raemistrasse 100, Zuerich, 8091, Switzerland

## Abstract

**Introduction:**

Maintaining arterial blood glucose within tight limits is beneficial in critically ill patients. Upper and lower limits of detrimental blood glucose levels must be determined.

**Methods:**

In 69 patients with severe traumatic brain injury (TBI), cerebral metabolism was monitored by assessing changes in arterial and jugular venous blood at normocarbia (partial arterial pressure of carbon dioxide (paCO_2_) 4.4 to 5.6 kPa), normoxia (partial arterial pressure of oxygen (paO_2_) 9 to 20 kPa), stable haematocrit (27 to 36%), brain temperature 35 to 38°C, and cerebral perfusion pressure (CPP) 70 to 90 mmHg. This resulted in a total of 43,896 values for glucose uptake, lactate release, oxygen extraction ratio (OER), carbon dioxide (CO_2_) and bicarbonate (HCO_3_) production, jugular venous oxygen saturation (SjvO_2_), oxygen-glucose index (OGI), lactate-glucose index (LGI) and lactate-oxygen index (LOI). Arterial blood glucose concentration-dependent influence was determined retrospectively by assessing changes in these parameters within pre-defined blood glucose clusters, ranging from less than 4 to more than 9 mmol/l.

**Results:**

Arterial blood glucose significantly influenced signs of cerebral metabolism reflected by increased cerebral glucose uptake, decreased cerebral lactate production, reduced oxygen consumption, negative LGI and decreased cerebral CO_2_/HCO_3 _production at arterial blood glucose levels above 6 to 7 mmol/l compared with lower arterial blood glucose concentrations. At blood glucose levels more than 8 mmol/l signs of increased anaerobic glycolysis (OGI less than 6) supervened.

**Conclusions:**

Maintaining arterial blood glucose levels between 6 and 8 mmol/l appears superior compared with lower and higher blood glucose concentrations in terms of stabilised cerebral metabolism. It appears that arterial blood glucose values below 6 and above 8 mmol/l should be avoided. Prospective analysis is required to determine the optimal arterial blood glucose target in patients suffering from severe TBI.

## Introduction

Traumatic brain injury (TBI) induces a plethora of structural and functional alterations, which contribute to subsequent deterioration as observed under clinical and experimental conditions. These changes occur in parallel and sequentially. They are associated with metabolic and energetic disturbances [1,2], due to impaired perfusion [3]; increased glycolysis [4] with increased lactate production [5]; regionally altered glucose uptake [6] and impaired glucose metabolism due to changes in enzymatic and mitochondrial activity [6-10]; functional derangements as observed in cortical spreading depolarisations (CSD) [11]; excitotoxicity with disturbed ionic homeostasis and activated intracellular destructive secondary cascades [12]; and increased activity of neurons and astrocytes [13]. These changes are not only restricted to the area of impact but are also observed in areas distant to the primary impact corresponding to contre-coup lesions [14].

Apart from local alterations systemic influences, such as hypotension, hypoxia and anaemia, are detrimental as cerebral oxygenation becomes insufficient. Consequently, induced pathological alterations are aggravated. In addition to hypotension, hypoxia and anaemia, changes in blood glucose levels induce additional damage. In this context, hyperglycaemia induces local acidosis [15,16] and oxidative stress, promotes oedema formation, impairs nitric oxide-mediated vasodilatation [17] and activates inflammation as reflected by increased leucocyte infiltration [18]. Hypoglycaemia increases glutamate release [19], induces metabolic impairment [19], and promotes generation of CSD which, in turn, generates and aggravates exisiting oedema [20].

In contemporary intensive care treatment of patients with severe TBI secondary brain damage must be avoided. In this context, hypoglycaemic as well as hyperglycaemic episodes need to be prevented. Although the upper limit of 10 mmol/l is well defined because hyperglycaemia exceeding 10 mmol/l is associated with increased mortality [21], the lower acceptable limit void of any damaging effect is still unclear. As pointed out by Strong and colleagues blood glucose levels less than 5 mmol/l increase the development of CSD [20]. In addition, maintaining blood glucose levels between 3.5 and 6.5 mmol/l increases frequency of hypoglycaemic episodes [22-27] and has been shown to induce metabolic impairment in brain-injured patients [19]. Thus, it appears that arterial blood glucose levels from 5 to less than 10 mmol/l could be more appropriate in terms of improved metabolic stability. The optimal limits, however, remain to be determined.

Intentionally lowering blood glucose levels and inducing hypoglycaemia to investigate the impact of different arterial blood glucose levels on cerebral metabolism following severe TBI is unethical in humans. In this context, retrospective analysis in evaluating a concentration-dependent impact of different arterial blood glucose concentrations on cerebral metabolism is helpful. For this, changes in various parameters of cerebral metabolism (jugular venous oxygen saturation (SjvO_2_), oxygen-glucose index (OGI), lactate-oxygen index (LOI), lactate-glucose index (LGI), arterio-jugular venous glucose and lactate differences) were determined for pre-defined arterial blood glucose values in a total of 69 patients with severe TBI. In addition, *post hoc *analysis of influence of time, different lesions, side of jugular venous catheter insertion and outcome was performed.

## Materials and methods

Following approval from the local Ethics Committee which waived the need for written informed consent for this retrospective analysis, patient records from a total of 69 patients treated on the intensive care unit (ICU) of the University Hospital Zuerich, Switzerland, from 2004 to 2006 were reviewed. All patients were required to have received a jugular venous catheter with a minimum monitoring time of 24 hours. Patients with severe injuries anticipated to succumb to their injuries within the first 24 hours were not considered for the present analysis. Barbiturates and propofol are known to dose-dependently suppress neuronal activity and cerebral metabolism [28]. To avoid difficult interpretation of the cerebral metabolic parameters due to differing depth of sedation only patients subjected to continuous infusion of fentanyl (Sintenyl; SINTETICA SA Pharmaceuticals, Switzerland) and midazolam (Dormicum; F. Hoffmann-La Roche AG, Switzerland) were investigated in the present study.

### Standardised treatment protocol

Following severe TBI, intubated and ventilated patients were treated according to our standardised treatment protocol. Following computed tomography (CT) diagnostic and surgical interventions including insertion of an intracranial pressure (ICP) probe (Neurovent, Raumedic AG, 95205 Münchberg, Germany) patients were transferred to our ICU. Continuous analgesia and sedation was controlled by bispectral index electroencephalography (BIS EEG; BIS VISTA, Aspect Medical Systems, Inc., One Upland Road, Norwood, MA, USA) tapering drug dosage to maintain a BIS level between 20 and 40. Noradrenaline, dobutamine and volume (crystalloids and colloids) were administered to maintain cerebral perfusion pressure (CPP) above 70 mmHg.

Sonographically guided insertion of a jugular bulb catheter in the larger internal jugular vein was performed within the first hour after admission to the ICU. In 88% of the investigated patients the right jugular vein was larger, irrespective of the type of lesion and predominant location of the brain lesions (Table 1). Subsequent radiological control using conventional x-ray of the lateral aspect of the cervical spine and head revealed the position of the tip of the jugular catheter. Whenever required the jugular catheter was repositioned with the tip of the catheter aimed at the caudal aspect of the mastoid process to avoid obstructing the jugular bulb and the sigmoid sinus. Thereafter, arterial and simultaneously drawn jugular venous blood samples were routinely investigated in four- to six-hour intervals. This sampling frequency was the same for every day and every patient until removal of the jugular venous catheter. Arterial and jugular venous blood gas analyses using commercially available pre-heparinised syringes (safe PICO Aspirator, Radiometer, Copenhagen; Radiometer Medical ApS, Åkadevej 21, DK-2700 Brønshøj, Denmark) were performed using the ABL825 Flex Analyzer (Radiometer Medical ApS, Åkadevej 21, DK-2700 Brønshøj, Denmark).

**Table 1:** Demographic data of 69 patients suffering from severe traumatic brain injury.

**Parameters**	**Median, range or %**
**Age (years)**	38, 18 to 65

**Gender**	76% male

**Initial GCS**	11, 3 to 15

**ISS**	34, 12 to 54

**Isolated TBI**	25%

**Mortality (%)**	26%

**Isolated lesions (%)**	32%
**EDH**	2%
**SDH**	8%
**Contusions**	12%
**tSAH**	4%
**Oedema**	6%

**Mixed lesions (%)**	68%

**Predominant side of brain lesion**	
**Right**	22%
**Left**	11%
**Bilateral**	67%

**Cannulation of right jugular vein**	
**Right-sided lesions**	13, 87%
**Left-sided lesions**	7, 88%
**Bilateral lesions**	42, 91%

**Length of ICU (days)**	
**Survivors**	16, 2 to 52
**Deceased**	10, 2 to 43

**Duration of jugular bulb (days)**	
**Survivors**	10, 2 to 24
**Deceased**	7, 2 to 15

EDH = epidural haematoma; GCS = Glasgow Coma Scale; ICU = intensive care unit; ISS = injury severity score; SDH = subdural haematoma; TBI = traumatic brain injury; tSAH = traumatic subarachnoid haemorrhage.

Differentiated CPP and ventilation management was guided by SjvO_2 _maintaining SjvO_2 _above 60%. Brain temperature was maintained between 35 and 36.0°C using cooling blankets or an intravenous cooling system (CoolGard3000; Alsius; 15770 Laguna Canyon Road, Suite 150, Irvine, CA, USA).

Overall, treatment measures were adapted and tapered to primarily maintain ICP below 15 mmHg. Following optimisation of therapeutic interventions an ICP below 20 mmHg was tolerated as long as CPP was maintained and cerebral metabolism was stable.

Patients received enteral nutrition via gastric or jejunal tube started within the first 12 hours. Administered calories were adapted according to indirect calorimetry performed twice weekly.

### Control and standardized management of arterial blood glucose concentrations

Arterial blood glucose was controlled in one- to four-hour intervals depending on the actual arterial blood glucose level determined in the arterial blood gas analysis. Arterial blood glucose target was set at 3.5 to 6.5 mmol/l based on the findings by van den Berghe and colleagues [22-24]. Arterial blood glucose was decreased by increasing insulin dose which was infused continuously. Arterial blood glucose was increased by decreasing infused insulin and by augmenting enteral nutrition. Glucose was not routinely infused as performed by van den Berghe and colleagues [22-24] to reduce the risk of promoting brain oedema formation. Transient glucose infusion was only considered in cases of severe hypoglycaemia (< 2 mmol/l) which occurred once in one patient.

### Calculated parameters of cerebral metabolism

#### Arterio-jugularvenous differences

Uptake and release of glucose (glc) and lactate (lac) can be assessed by calculating corresponding arterio-jugularvenous differences (AJVD). Although positive values reflect uptake, negative values unmask cerebral release:

AJVD glc = arterial glc - jugularvenous glc

AJVD lac = arterial lac - jugularvenous lac

#### Cerebral arterio-jugularvenous difference in oxygen

Arterio-jugularvenous difference in oxygen (avDO_2_) was calculated based on the arterial (caO_2_) and jugular venous oxygen (cjvO_2_) content:

avDO_2 _= caO_2 _- cjvO_2_

Arterial and jugular venous oxygen content were calculated based on haemoglobin (Hb) concentration and oxygen saturation in arterial (SaO_2_) and jugular venous (SjvO_2_) blood using the following equations:

caO_2 _= (1.34 × Hb × SaO_2_) + (0.003 × paO_2_)

cjvO_2 _= (1.34 × Hb × SjvO_2_) + (0.003 × pjvO_2_)

(paO2 = partial arterial oxygen tension; pjvO2 = partial jugular venous oxygen tension)

#### Oxygen extraction rate

Oxygen extraction ratio (OER) was calculated based on the equation:

OER = (caO_2 _- cjvO_2_)/caO_2_, expressed in %.

#### Oxygen-glucose index

OGI was calculated based on changes in avDO_2 _and AJVD glc:

OGI = avDO_2_/AJVD glc

During aerobic glycolysis approximately six molecules of oxygen are used to oxidate one molecule of glucose. Whenever glucose metabolism exceeds oxygen consumption, the calculated OGI will be less than 6, thereby reflecting anaerobic glycolysis. An OGI of more than 6 indicates aerobic metabolism of substrates other than glucose, such as lactate.

#### Lactate-glucose index

LGI was calculated considering changes in AJVD lac and AJVD glc:

LGI = AJVD lac/AJVD glc

LGI reflects generation of cerebral lactate from glucose. Increased cerebral lactate production results in negative LGI values, while positive LGI reflects lactate uptake.

#### Lactate-oxygen index

LOI was calculated using the following equation:

LOI = AJVD lac/avDO_2_

LOI can be used as a crude estimate for the extent of cerebral anaerobic metabolism relative to oxidative metabolism. In this context, lactate release results in negative LOI although lactate uptake is reflected by a positive LOI.

#### Arterio-jugularvenous difference in pH

AJVD pH can be used to assess dynamic changes. Less positive values unmask decreased release of H^+ ^ions, reduced production of carbon dioxide (CO_2_) or sustained buffering of acidosis due to increased release of bicarbonate (HCO_3_).

AJVD pH = pHa - pHjv

#### Arterio-jugularvenous difference in pCO_2_

Negative AJVD partial pressure of CO_2 _(pCO_2_) values represent increased cerebral production of CO_2_. Less negative AJVD pCO_2 _values unmask reduced release of CO_2:_

AJVD pCO_2 _= paCO_2 _- pjvCO_2_

where paCO2 = partial arterial pressure of carbon dioxide and pjvCO2 = partial jugular venous pressure of carbon dioxide.

#### Arterio-jugularvenous difference in HCO_3_

Dynamic changes in AJVD HCO_3 _reflect production of HCO_3 _and intracerebral buffer capacity. In this context, negative AJVD HCO_3 _represent increased HCO_3 _production.

AJVD HCO_3 _= arterial HCO_3 _- jugularvenous HCO_3_

#### Detailed evaluation

Pre-defined parameters (ICP, CPP, paCO_2_, parameters of cerebral metabolism) were assessed for different arterial blood glucose values grouped in 1 mmol/l clusters ranging from less than 4 to more than 9 mmol/l.

Parameters of cerebral metabolism were investigated under conditions of normocarbia (paCO_2 _4.4 to 5.6 kPa), normoxia (paO_2 _9 to 20 kPa) and with a haematocrit between 27 and 36%. In addition, only values determined at a temperature between 35 and 38°C and CPP between 70 and 90 mmHg were considered. This resulted in a total of 3658 values per investigated parameter, representing 69% of all recorded time points. When considering the influence of arterial blood glucose clusters the remaining values per defined cluster were too small to allow meaningful statistical analysis.

Time dependency was determined by evaluating changes of the pre-defined paramaters within the arterial blood glucose clusters during the first, second and third week.

Lesion-dependent influences were assessed by comparing the pre-defined parameters between lesion subtypes: isolated lesions versus mixed lesions. A more detailed analysis was not possible due to a limited number of patients and samples per lesion subgroup.

Influence of outcome was determined by grouping the pre-defined parameters according to survivors and non-survivors.

Calculation of frequency of pathological values allow to determine the impact of different arterial blood glucose clusters on cerebral brain metabolism. For this, frequency of SjvO_2 _less than 60%, OGI less than 6, negative LGI, negative LOI and negative AJVD lactate levels reflecting increased cerebral oxygen consumption (SjvO_2_, OER), anaerobic glycolysis (OGI) and lactate production (LGI, LOI, AJVD lactate) were assessed in pre-defined arterial blood glucose clusters.

### Statistical analysis

Graphical and statistical analysis was performed using SigmaPlot 10.0 and SigmaStat 3.5, (Systat Software, Inc. San Jose, CA., USA) respectively. Changes over time and between groups were evaluated for statistically significant difference using the Mann Whitney rank sum test and analysis of variance (ANOVA) on ranks with *post hoc *all pairwise multiple comparison procedures (Dunn's test). Differences were rated significant with a p < 0.05.

## Results

### Demographic data

Demographic data of the investigated 69 patients are given in Table 1. These patients reflect the population of patients with severe TBI treated at our institution.

### Number of determined values

Overall, a total of 3658 arterial and corresponding jugular venous blood gas analyses were performed, resulting in a total of 43,896 values for the 12 pre-defined parameters (ICP, CPP, paCO_2_, SjvO_2_, OER, LOI, LGI, OGI, AJVD glc, AJVD lac, AJVD pH, AJVD CO_2 _and AJVD HCO_3_).

According to the individual clinical courses, the majority of values were determined in the first week (52%), followed by 39% in the second week and 9% in the third week. Thus, the strongest statistical power is found during the first two weeks.

### Relative frequency of arterial blood glucose values

**Figure 1:**
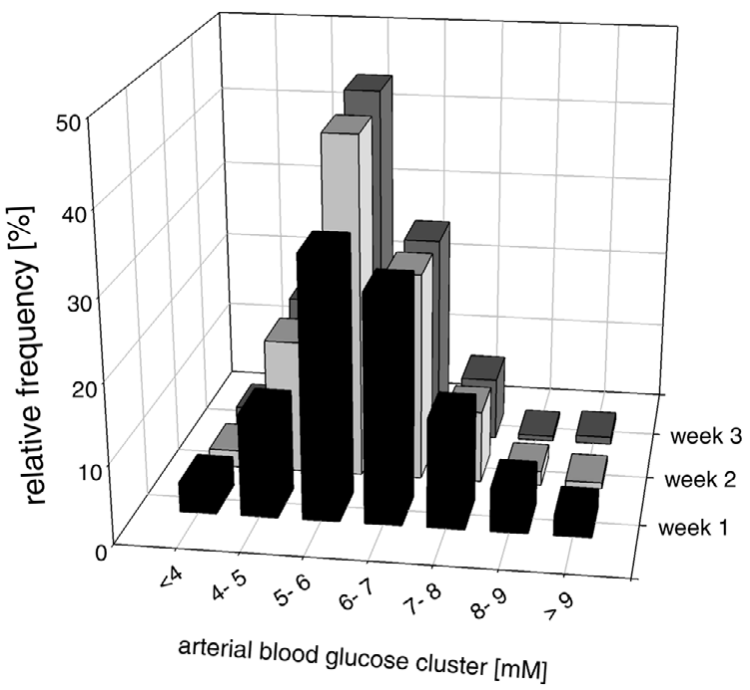
Changes in relative frequency of arterial blood glucose levels determined in pre-defined blood glucose clusters for the first three weeks in a total of 69 patients. The majority of arterial blood glucose values were found between 5 and 6 mmol/l and 6 and 7 mmol/l, which was similar at all investigated time points.

During weeks one to three, arterial blood glucose concentrations were predominantly measured between 5 and 6 and 6 and 7 mmol/l corresponding to the set blood glucose target of 3.5 to 6.5 mmol/l (Figure 1). There was no significant difference between weeks one and three.

### Glucose-dependent and time-dependent changes

Pre-defined arterial blood glucose clusters resulted in different numbers of values per cluster for the different parameters: less than 4 mmol/l = 111; 4 to 5 mmol/l = 543; 5 to 6 mmol/l = 1385; 6 to 7 mmol/l = 1005; 7 to 8 mmol/l = 418; 8 to 9 mmol/l = 134; more than 9 mmol/l = 62 values.

**Figure 2:**
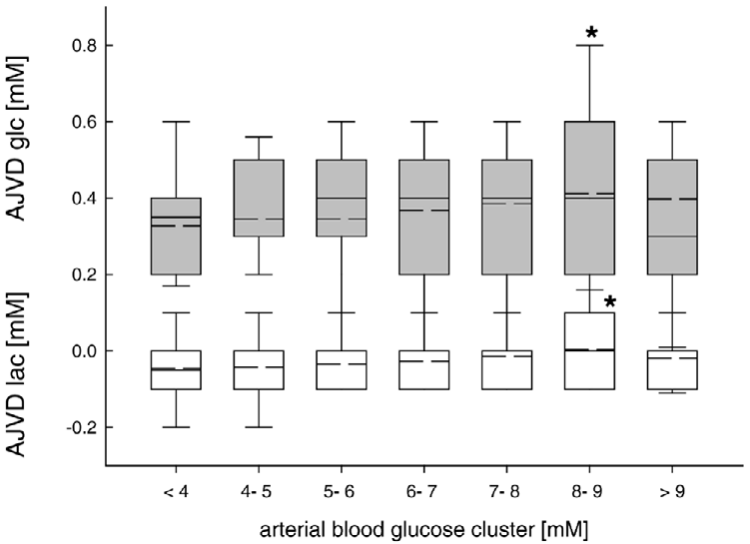
Changes in calculated arterial jugular venous differences in glucose (grey box plots) and lactate (white box plots) for pre-defined arterial blood glucose clusters. Compared with low arterial blood glucose levels cerebral glucose uptake was significantly increased although cerebral lactate production was significantly decreased at arterial blood glucose concentrations between 7.5 and 8.5 mmol/l (*p < 0.001; analysis of variance (ANOVA) on ranks and *post hoc* Dunn's test). Cerebral uptake is reflected by positive values although cerebral release is unmasked by negative values. AJVD = arterio-jugularvenous difference; glc = glucose; lac = lactate.

Increasing arterial blood glucose was associated with a significantly increased cerebral glucose uptake reflected by a more positive AJVD glc (Figure 2). In parallel, lactate release was decreased revealed by a less negative AJVD lac approaching positive values (Figure 2). Significant increases were observed at arterial blood glucose levels between 8 and 9 mmol/l compared with arterial blood glucose less than 8 mmol/l. There were no significant differences between the different weeks (weeks 1, 2 and 3) (data not shown).

**Figure 3:**
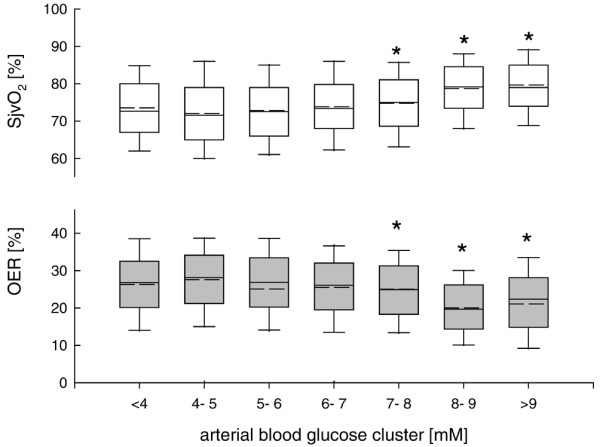
Changes in jugular venous oxygen saturation (SjvO_2_) (white box plots) within pre-defined arterial blood glucose clusters. Changes in SjvO2 were significantly increased at arterial blood glucose levels above 8 mmol/l compared with lower arterial blood glucose levels. *p < 0.001; analysis of variance (ANOVA) on ranks and post hoc Dunn's test. Calculated oxygen extraction ratio (OER) (grey box plots) within pre-defined arterial blood glucose clusters was significantly decreased at arterial blood glucose levels above 8 mmol/l compared with lower arterial blood glucose levels. *p < 0.001; ANOVA on ranks and post hoc Dunn's test.

OER was significantly decreased reaching lowest values at blood glucose more than 8 mmol/l (Figure 3). Changes in OER were reflected by increased SjvO_2 _levels, respectively, reaching highest values at blood glucose more than 8 mmol/l (Figure 3).

**Figure 4:**
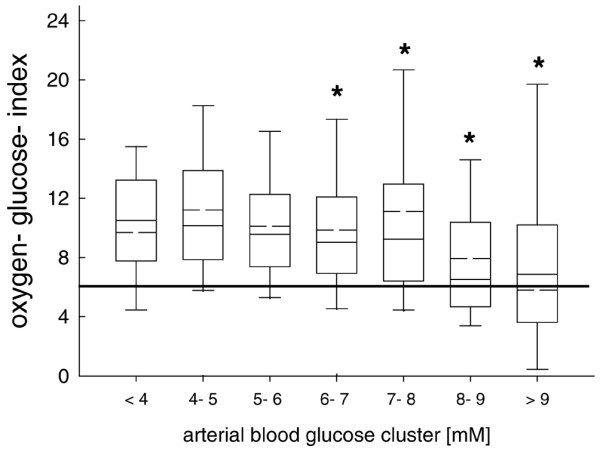
Oxygen-glucose index (OGI) was significantly increased compared with normal values. This is reflected by the straight line at 6. With increasing arterial blood glucose concentrations OGI was significantly decreased compared with lower arterial blood glucose levels. *p < 0.001; analysis of variance (ANOVA) on ranks and *post hoc *Dunn's test.

Calculated OGI was significantly decreased with increasing arterial blood glucose levels exceeding 8 mmol/l (Figure 4). There was no difference over time (data not shown).

Calculated LOI showed a trend towards elevated values with increasing arterial blood glucose levels. There was no difference over time (data not shown).

**Figure 5:**
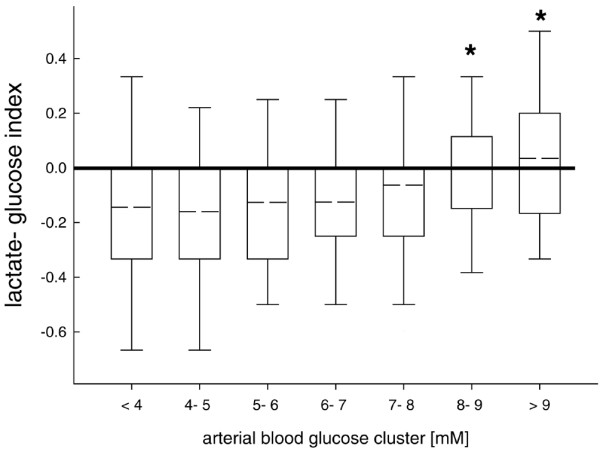
Lactate-glucose-index (LGI) was significantly decreased compared with normal values. With arterial blood glucose levels exceeding 8 mmol/l, LGI was significantly increased reaching normal values. *p < 0.001; analysis of variance (ANOVA) on ranks and *post hoc *Dunn's test.

Calculated LGI approached positive values and was significantly increased with higher arterial blood glucose concentrations exceeding 8 mmol/l (Figure 5). There was no difference over time (data not shown).

**Figure 6:**
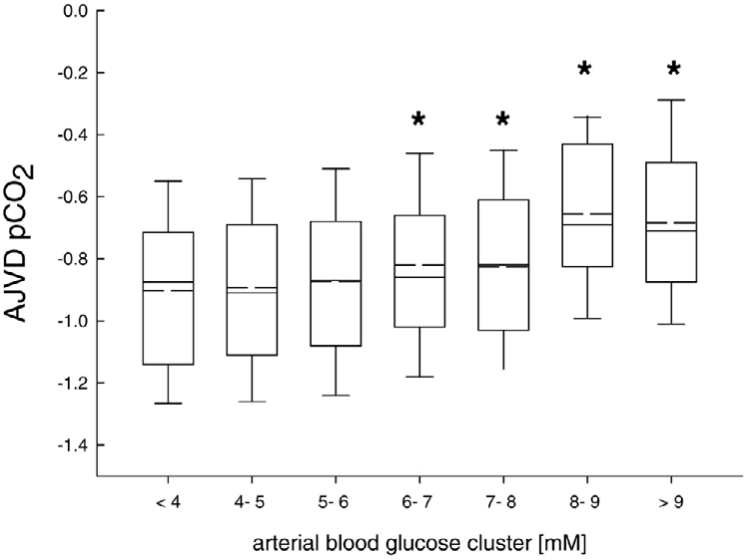
Calculated arterial jugular venous difference in partial pressure of carbon dioxide (AJVDpCO_2_). Values were significantly increased with arterial blood glucose concentrations exceeding 6 mmol/l compared with low arterial blood glucose levels. *p < 0.001; analysis of variance (ANOVA) on ranks and *post hoc* Dunn's test.

**Figure 7:**
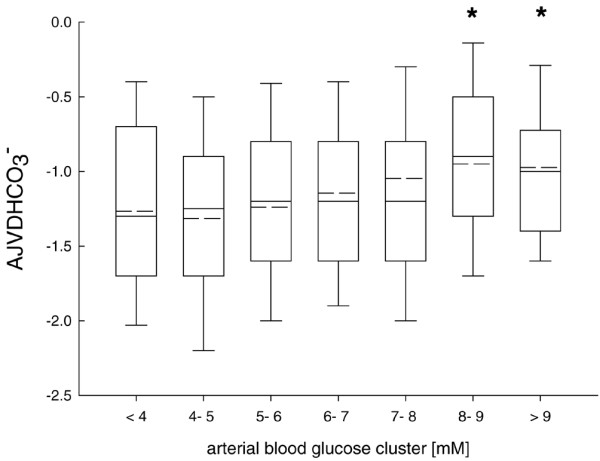
Calculated arterial jugular venous difference in bicarbonate (AJVDHCO_3_^-^). Values were significantly increased with arterial blood glucose concentrations exceeding 8 mmol/l compared with low arterial blood glucose levels. *p < 0.001; analysis of variance (ANOVA) on ranks and *post hoc* Dunn's test.

Cerebral release/production of CO_2 _and HCO_3 _was significantly reduced with arterial blood glucose exceeding 6 mmol/l (Figures 6 and 7). This was reflected by a smaller AJVD pH (data not shown).

**Figure 8:**
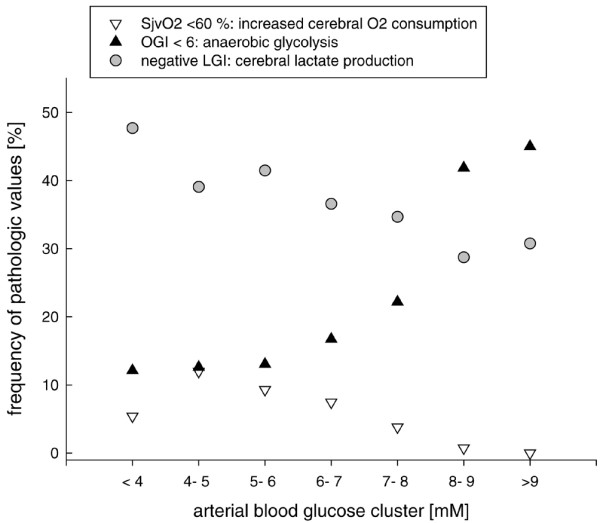
Calculation of frequency of pathological values within pre-defined arterial blood glucose clusters. Values are given for increased cerebral oxygen consumption (jugular venous oxygen saturation (SjvO2) less than 60%), sustained anaerobic glycolysis (oxygen-glucose index (OGI) less than 6), and increased cerebral lactate production (negative lactate-glucose index (LGI)). With elevated arterial blood glucose the rate of increased cerebral oxygen consumption (SjvO2 less than 60%) was reduced which coincided with decreased rate of increased cerebral lactate production (negative LGI). However, frequency of anaerobic glycolysis (OGI less than 6) was increased.

With elevated arterial blood glucose levels, frequency of increased cerebral oxygen consumption (SjvO_2 _less than 60%) and cerebral lactate production (negative LGI values) were reduced (Figure 8). Rate of anaerobic glycolysis (OGI less than 6), however, was increased at higher arterial blood glucose levels (Figure 8).

Different blood glucose levels did not influence ICP and CPP values (data not shown).

### Lesion-dependent changes

Pre-defined cerebral metabolic parameters, as well as ICP and CPP, were similar in patients with isolated lesions compared with mixed lesions (data not shown). There were no differences in type and extent of therapeutic measures.

### Side-dependent changes

In the majority of the investigated patients (62 of 69 patients) the jugular venous catheter was inserted in the right jugular vein, irrespective of the type of lesion and the predominant side of the brain lesions (Table 1) (right-sided brain lesions: 13 of 15 patients; left-sided brain lesions: 7 of 8 patients; bilateral brain lesions: 42 of 46 patients). In the remaining seven patients, the left jugular vein was canulated. There was no difference in brain metabolism between left-sided or right-sided cannulation. However, the low number of patients and uneven distribution within the different brain lesions did not allow statistical analysis.

### Outcome-dependent changes

Investigated metabolic parameters could not differentiate non-surviving patients from surviving patients (data not shown).

## Discussion

Bed-side analysis of changes in arterial and jugular venous differences and their derived indices of cerebral metabolism differentiated less favourable from more favourable arterial blood glucose concentrations. Overall, cerebral metabolism appeared more stable as judged by increased glucose uptake, reduced cerebral lactate, CO_2 _and HCO_3 _production/release, elevated SjvO_2_, decreased OER, increased LOI and elevated LGI with arterial blood glucose levels between 8 and 9 mmol/l.

### Limitations of the study

The retrospective nature of the present analysis does not allow the clear definition of the dynamic processes induced by specific arterial blood glucose concentrations or induced therapeutic interventions because investigated changes were taken from the pooled data obtained in 69 patients with severe TBI. These pooled data consist of arterial and jugular venous blood samples which were drawn at fixed time intervals predominantly ranging from four to six hours. These time intervals were independent of changes in arterial blood glucose values which might have occurred between these sampling intervals. A prospective study designed to specifically investigate the impact of dynamic changes by assessing alterations of cerebral metabolic parameters at pre-defined changes in arterial blood glucose levels is required to address this issue.

Calculated differences and indices of cerebral metabolism are widely accepted to gain insight in otherwise occult changes within the brain [7,29-33]. However, the low temporal and spatial resolution limit any detailed information concerning changes between the individual blood sampling time points and influences of injured versus non-injured or lesser injured tissue because blood samples were drawn in four- to six-hour intervals and jugular venous blood reflects global rather than local intracerebral changes. In this context it has been shown that SjvO_2 _only partially reflects pathological intracerebral alterations [34] which show a strong regional heterogeneity within peri-lesional tissue compared with lesions [35].

As observed in healthy volunteers magnetic resonance venography revealed a significant asymmetry in the venous blood flow from the superior sagittal sinus flow to one transverse sinus in 84% of the volunteers [36]. Based on a theoretical model this is accepted to result in an asymmetry in jugular venous oxygen saturation measurements in patients with a supratentorial lesion [36]. As shown by Metz and colleagues [37], monitoring of cerebral metabolism using bilateral jugular venous catheters is superior to the unilateral approach when searching for signs of posttraumatic cerebral ischaemia due to insufficient CPP and hyperventilation. Nevertheless 87% of ischaemic events were detected when monitoring ipsilateral to the predominant lesion or the side with the predominant jugular venous outflow (in patients with diffuse brain injury). Thus, we can expect to unmask pathological alterations in the majority of our patients. The scientific superiority of bilateral cannulation of the jugular vein is off-set by the clinically relevant increased risk of bilateral thrombosis formation, which could result in increased ICP because of reduced venous outflow.

Although microdialysis [1,2,5,16,19,38,39], positron emission tomography (PET) [4-6,39,40], and single photon emission computed tomography (SPECT) [41] allow more detailed insight, these techniques are also confronted with specific limitations, such as high costs, decreased regional and temporal resolution, respectively.

Continuous arterial and jugular venous blood sampling with subsequent analysis of metabolic parameters would be helpful as described under experimental conditions [42]. However, appropriate techniques have not yet been developed for the clinical application of this. Until then, easy and cheap analysis of intermittently drawn blood gases which is an integral part of contemporary intensive care treatment of critically ill patients is the only feasible approach applicable in any specialised ICU.

### Glucose and cerebral metabolism

Glucose is the predominant fuel for energy consuming processes within the brain [32]. Glucose is mainly used by the Na^+^/K^+ ^ATPase which is indispensable to maintain membrane stability and prevent functional, as well as structural, cell damage [43]. Various endothelial, glial and neuronal glucose transporters with different transport characteristics guarantee sufficient glucose transport across the blood brain barrier (GLUT1) as well as glial (GLUT1, 5) and neuronal (GLUT3, 4, 6, 8) glucose uptake [13,44]. In this context, the neuronal GLUT3 exhibits a lower K_m _(Michaelis constant) and a higher V_max _(maximal transport velocity) compared with the other glucose transporters: K_m _= 1.4 to 2.8 mmol/l [13,44], V_max _= 5 to 34.6 nM/10^6 ^cells/minute [13], resulting in a significantly higher affinity and transport capacity compared with GLUT1, for example. These characteristics, in turn, guarantee adequate neuronal glucose utilisation under conditions of decreased glucose supply. This is important because the ambient glucose levels within the neuronal environment is rather low ranging from 1 to 2 mmol/l compared with normal blood glucose levels between 5 and 6 mmol/l. Thus, any decrease in arterial blood glucose in conjunction with impaired endothelial glucose transport due to reduced GLUT1 expression will endanger neuronal function and viability.

Following TBI, increased GLUT3 expression [45] guarantees neuronal glucose uptake although decreased GLUT1 expression [46], as found under experimental conditions, limits endothelial glucose transport. Decreased GLUT1 expression in conjunction with reduced blood glucose levels result in a concentration-dependent decrease in glucose flux, which is mostly sustained at blood glucose levels below 3 mmol/l [47].

Under clinical conditions it is unclear which changes in presence and function of the different GLUT subtypes are prevalent. The arterial blood glucose concentration-dependent cerebral uptake of glucose, as seen in the present study, suggests that arterial blood glucose concentrations maintained below the optimal K_m _of the endothelial GLUT1, that is less than 8 mmol/l [13,44] will result in insufficient supply. This can be overcome by maintaining arterial blood glucose levels at about 8 mmol/l as reflected by increased metabolic stability. This is in line with findings showing the impact of decreased glucose supply on posttraumatic functional disturbances after TBI in terms of induced CSD [20], increased extracellular glutamate and elevated lactate/pyruvate ratio [19]. As pointed out by Vespa and colleagues, cerebral oxygen consumption was decreased in patients with higher blood glucose concentrations (120 to 150 versus 90 to 120 mg/dl) [19]. This is also seen in the present patients. These findings strongly suggest that activation of glucose transporter systems influence cerebral oxygen consumption. As shown by Abate and colleagues [48] increased cerebral glucose consumption is associated with elevated OER, although low cerebral glucose consumption results in decreased OER. Thus, the present data suggest that metabolic instability, which can also occur independently from cerebral ischaemia [2], can be influenced substantially by changing arterial blood glucose levels.

Classically, OER has always been discussed in the context of altered cerebral perfusion due to hypotension and hyperventilation [49] as increased OER reflects insufficient cerebral perfusion. The present findings, in conjunction with previously published data [19,45-48], suggest that changes in glucose metabolism substantially influence OER. Increased oxygen consumption resulting from energetic impairment (lactate production, elevated lactate/pyruvate ratio) and neuronal excitation due to sustained glutamate release could increase cerebral perfusion to correct this oxygen and energy deficit. This, however, requires an intact coupling between metabolism and perfusion [50], which is known to be disturbed after severe TBI [34] and during sedation/anaesthesia [51].

Based on the facts that CPP was maintained above 70 mmHg and sedation was unchanged at all investigated time points of analysis – assuming sufficient regional cerebral perfusion and stable pharmacological coma – we speculate that metabolic impairment due to low arterial blood glucose levels was the driving force for the observed increase in OER at arterial blood glucose values less than 7 mmol/l coinciding with increased lactate and CO_2 _production, and lower LGI. However, the present data do not allow for the reliable differentiation of whether metabolic impairment might aggravate oedema formation resulting in microcirculatory deterioration which, in turn, increases OER. It also remains unclear whether the increased CO_2 _production reflected by the negative AJVD pCO_2 _counteracts perfusion, metabolism mismatch or contributes to ongoing metabolic impairment due to vessel dilation and subsequent sustained brain swelling with compression of the microcirculation. Further analysis including assessment of cerebral perfusion is warranted to determine the functional impact of the metabolic changes observed in the present retrospective analysis.

### Signs of tissue acidosis and regulation of cerebral metabolism

Severe TBI induces brain tissue acidosis reflected by significantly decreased brain tissue pH inversely correlated with elevated brain lactate and pCO_2 _during the first posttraumatic day [52]. Brain pH can either be determined directly by inserting specialised probes to measure pH or indirectly by measuring tissue pCO_2 _and lactate. In addition, assessing changes in arterio-jugular venous differences in pCO_2 _as reported by Chieregato and colleagues [33] or HCO_3_^- ^as performed in the present study can be used to indirectly determine changes in brain pH. Apart from insufficient cerebral perfusion and cerebral oxygenation [52] concomitant hyperglycaemia has also been shown to aggravate TBI- and ischaemia-induced brain tissue acidosis [53,54]. Sustained cerebral CO_2 _and HCO_3_^- ^production due to increased metabolism reflect underlying activation of various transporter systems and regulatory mechanisms. In this context, cerebral pH is regulated by Na^+^/H^+ ^exchange, Na+-driven Cl^-^/HCO_3_^- ^exchange, Na^+^-HCO_3_^- ^cotransport and passive Cl^-^/HCO_3_^- ^exchange [55].

As unmasked by the present study, low arterial blood glucose levels less than 8 mmol/l are also associated with sustained CO_2_, HCO_3_^- ^and lactate production suggesting that inadequate glucose supply activates various transporter systems such as the Na^+^/K^+^ATPase and glucose transporters to meet increased metabolic and energetic demands resulting from, for example, sustained hypoglycaemia-induced glutamate release [19] and subsequent glutamate-mediated increased cerebral glucose consumption [56].

### Glucose and secondary cerebral damage

Any decrease in arterial glucose will impair cerebral glucose-dependent pathways, thereby resulting in disturbed metabolism as reflected by increased lactate/pyruvate ratio [19]. This, in turn, can induce excitotoxic damage resulting in increased extracellular glutamate levels [19]. As shown by Shulman and colleagues [57] approximately 80% of cortical glucose consumption (in the rat brain) is driven by glutamate cycling. Thus, glutamate release due to reduced arterial blood glucose levels [19] increases cerebral glucose utilisation, which cannot be met if arterial blood glucose remains low. This is of importance whenever glucose uptake, glucose metabolism, enzymatic function, local perfusion and local diffusion processes are disturbed. Together with these alterations reduced blood glucose can aggravate underlying brain damage. In this context, a decrease in blood glucose levels below 8 mmol/l was associated with a significant elevation in peri-ischaemic cortical depolarisations [58]. This coincided with metabolic impairment reflected by an increase in extracellular cerebral lactate and decrease in extracellular glucose measured by microdialysis [58]. The occurrence of cortical depolarisations was dramatically increased when blood glucose levels dropped below 6 mmol/l [58,59]. Consequently, induction of CSD known to promote secondary damage can be avoided by maintaining arterial blood glucose above 5 mmol/l. As suggested by the present findings arterial blood glucose ranging from 7 to 9 mmol/l appear more beneficial in terms of improved metabolic stability.

### Which arterial blood glucose concentration is optimal after severe TBI?

Under conditions of relative cerebral glucose insufficiency due to increased cerebral glycolysis or absolute glucose insufficiency caused by systemic hypoglycemia, the brain can metabolize lactate, pyruvate, and keton bodies [13,32]. However, lactate metabolism is less efficient than glycolysis and mitochondrial oxidative phosphorylation which result in higher ATP production compared with lactate degradation. Lactate metabolism includes energy-consuming shuttling processes to transport lactate from astrocytes to neurons for subsequent generation of pyruvate via lactate dehydrogenase and further processing in the citric acid cycle and mitochondrial respiratory chain [13]. Although cerebral glycogen stores have been shown to exceed arterial blood glucose levels by three- to four-fold during euglycaemia in healthy controls [60], reflecting an additional valuable energetic reserve, it is unclear to what extent and for which duration glycogenolysis can fuel energy-requiring processes under pathological conditions. As suggested by Otori and colleagues, increase in cerebral glycogen content following experimental TBI could serve as an endogenous source of metabolic energy [61]. Thus, a decrease in arterial blood glucose should trigger glycogenolysis to maintain extracellular glucose concentrations and avoid metabolic impairment. However, this does not seem to occur under clinical conditions because decreased blood glucose resulted in a significant reduction in extracellular glucose concentrations determined by microdialysis in TBI and epileptic patients [19,62]. Hypoglycaemia-induced impaired cerebral metabolism in terms of lactate production, as observed in the present study, and increased lactate/pyruvate ratio [19] reflects a subordinated importance of glycogen under these specific conditions to prevent energetic deterioration. Consequently, sufficient cerebral glucose supply must be guaranteed to prevent avoidable secondary brain damage.

As suggested by the present retrospective analysis, optimal arterial blood glucose levels range from 6 to 8 mmol/l. With arterial blood glucose levels exceeding 8 mmol/l differentiated metabolic pathways appear to be activated as reflected by decreased rate of increased oxygen consumption and reduced frequency of increased cerebral lactate production. At the same time, however, anaerobic glycolysis reflected by OGI values less than 6 was increased. Apart from anaerobic metabolism, which implies underlying ischaemia or hypoxia, it could be possible that non-oxidative metabolism resulting from mitochondrial damage and impaired oxidative phosphorylation despite sufficient perfusion and oxygen supply accounts for the observed decrease in OGI. This is in line with the pathophysiological mechanism of destructive influence of elevated arterial blood glucose levels on mitochondria due to hyperglycaemia-induced production of free oxygen radicals with subsequent impairment of oxidative phosphorylation as discussed by van den Berghe and colleagues [22-24].

Taken together, arterial blood glucose levels between 6 and 8 mmol/l could be an appropriate range for patients suffering from severe TBI.

## Conclusions

To avoid cerebral metabolic impairment and prevent secondary brain damage adequate blood glucose levels must be induced and maintained during the intensive care phase. Although substantial and reproducible evidence exists to avoid arterial blood glucose levels exceeding 10 mmol/l, the optimal lower blood glucose level is less clear. The present results strongly suggest that arterial blood glucose concentrations less than 6 mmol/l should be avoided and that optimal cerebral metabolic stability is found at arterial blood glucose levels of about 8 mmol/l as reflected by increased cerebral glucose uptake, decreased cerebral lactate production, increased SjvO_2_, as well as decreased cerebral oxygen extraction, CO_2 _and HCO_3 _production. The increased frequency of reduced OGI below 6 at arterial blood glucose levels exceeding 8 mmol/l appears to delineate the upper limit of acceptable arterial blood glucose levels. Prospective studies are needed to define the optimal arterial blood glucose target in patients with severe TBI.

## Key messages

• Changes in cerebral metabolism determined by analysing jugular venous blood gases and calculating arterial-jugular venous differences of metabolic indices are significantly influenced by arterial blood glucose concentrations.

• Arterial blood glucose concentration dependently improved cerebral metabolism reflected by elevated SjvO_2_, increased cerebral glucose uptake, decreased cerebral lactate production, reduced CO_2 _and HCO_3_^- ^production, and increased lactate-glucose index.

• Increased incidence in decreased OGI (< 6) reflecting anaerobic glycolysis occurred at arterial blood glucose more than 8 mmol/l but was not associated with increased cerebral lactate production.

• Cerebral metabolic stability is reached at arterial blood glucose levels between 6 and 8 mmol/l.

• Arterial blood glucose concentrations less than 6 and more than 8 mmol/l should be avoided to prevent signs of worsened cerebral metabolism reflected by increased cerebral lactate production and anaerobic glycolysis, respectively.

## Abbreviations

AJVD: arterio-jugular venous difference; ANOVA: analysis of variance; CO_2_: carbon dioxide; CPP: cerebral perfusion pressure; CSD: cortical spreading depolarisations; CT: computed tomography; HCO_3_: bicarbonate; ICP: intracranial pressure; ICU: intensive care unit; LGI: lactate-glucose index; LOI: lactate-oxygen index; OER: oxygen extraction ratio; OGI: oxygen-glucose index; PaCO_2_: partial arterial pressure of carbon dioxide; PCO_2_: partial pressure of carbon dioxide; PaO_2_: partial arterial pressure of oxygen; SjvO_2_: jugular venous oxygen saturation; TBI: traumatic brain injury.

## Competing interests

The authors declare that they have no competing interests.

## Authors' contributions

MH collected the majority of the data, drafted parts of the manuscript and performed graphical analysis. MB helped to analyse and interpret the data and drafted parts of the manuscript. SL and JS were responsible for data collection and maintaining the data bank. SRC, MK and RS helped analysing and interpreting the data. JFS conceived the study design, collected parts of the data, performed graphical and statistical analysis, and drafted parts of the manuscript.
